# The development of chronic cough in children following presentation to a tertiary paediatric emergency department with acute respiratory illness: study protocol for a prospective cohort study

**DOI:** 10.1186/1471-2431-13-125

**Published:** 2013-08-15

**Authors:** Benjamin J Drescher, Anne B Chang, Natalie Phillips, Jason Acworth, Julie Marchant, Theo P Sloots, Michael David, Kerry-Ann F O’Grady

**Affiliations:** 1 Queensland Children’s Medical Research Institute, The University of Queensland, Level 4, Foundation Building, Royal Children’s Hospital, Herston Road, Brisbane, Herston QLD 4029, Australia; 2 Queensland Children’s Medical Research Institute, Queensland University of Technology, Level 4, Foundation Building, Royal Children’s Hospital, Herston Road, Brisbane, Herston QLD 4029, Australia; 3 Queensland Children’s Respiratory Centre, Queensland Children’s Health Services, Royal Children’s Hospital, Herston Road, Brisbane, Herston QLD 4029, Australia; 4 Menzies School of Health Research, Charles Darwin University, Rocklands Drive, Darwin, Tiwi NT 0811, Australia; 5 Department of Emergency Medicine, Queensland Children’s Health Services, Royal Children’s Hospital, Herston Road, Brisbane, Herston QLD 4029, Australia; 6 Sir Albert Sakzewski Virus Research Centre, Queensland Paediatric Infectious Diseases Laboratory, Royal Children’s Hospital, Herston Road, Brisbane, Herston QLD 4029, Australia

**Keywords:** Acute respiratory illness, Chronic lung disease, Chronic cough, Paediatric emergency department

## Abstract

**Background:**

Acute respiratory illness, a leading cause of cough in children, accounts for a substantial proportion of childhood morbidity and mortality worldwide. In some children acute cough progresses to chronic cough (>4 weeks duration), impacting on morbidity and decreasing quality of life. Despite the importance of chronic cough as a cause of substantial childhood morbidity and associated economic, family and social costs, data on the prevalence, predictors, aetiology and natural history of the symptom are scarce. This study aims to comprehensively describe the epidemiology, aetiology and outcomes of cough during and after acute respiratory illness in children presenting to a tertiary paediatric emergency department.

**Methods/design:**

A prospective cohort study of children aged <15 years attending the Royal Children’s Hospital Emergency Department, Brisbane, for a respiratory illness that includes parent reported cough (wet or dry) as a symptom. The primary objective is to determine the prevalence and predictors of chronic cough (≥4 weeks duration) post presentation with acute respiratory illness. Demographic, epidemiological, risk factor, microbiological and clinical data are completed at enrolment. Subjects complete daily cough dairies and weekly follow-up contacts for 28(±3) days to ascertain cough persistence. Children who continue to cough for 28 days post enrolment are referred to a paediatric respiratory physician for review. Primary analysis will be the proportion of children with persistent cough at day 28(±3). Multivariate analyses will be performed to evaluate variables independently associated with chronic cough at day 28(±3).

**Discussion:**

Our protocol will be the first to comprehensively describe the natural history, epidemiology, aetiology and outcomes of cough during and after acute respiratory illness in children. The results will contribute to studies leading to the development of evidence-based clinical guidelines to improve the early detection and management of chronic cough in children during and after acute respiratory illness.

## Background

Acute respiratory illness (ARI) a leading cause of cough in children accounts for a substantial proportion of childhood morbidity and mortality worldwide [1-3]. Cough in children is one of the most common reasons for medical encounters both in Australia [4] and internationally [5,6]. In the UK, 30% of all paediatric primary care encounters are due to respiratory illnesses, with cough as a symptom accounting for over 8% of all medical presentations [6]. In the United States, cough as a symptom is the 4th leading reason for emergency department (ED) attendance across all ages, accounting for 3% of all presentations [5]. In a UK study designed to test the repeatability of a parent-completed respiratory questionnaire, one third of parents with young children reported cough in the absence of a cold whilst one in five parents reported cough at night [7].

Cough in children is symptomatic of a broad range of respiratory illnesses and infections ranging from mild and transient upper respiratory tract infections to serious chronic disease [8]. In some children acute cough can lead to chronic cough (defined as cough lasting >4 weeks), which may be the sole presenting symptom of an underlying respiratory illness. Parents of children with chronic cough typically seek five or more medical consultations prior to referral to respiratory specialists [9]. Despite chronic cough in children accounting for substantial direct and indirect costs for health service providers, patients and their families, it remains an under-recognised and inadequately researched cause of morbidity in children [9].

One of the constructs in the assessment of chronic cough in children is cough quality which includes dry and wet cough [10]. Chronic wet cough is a key symptom of protracted bacterial bronchitis (PBB), chronic suppurative lung disease (CSLD) and bronchiectasis in children and if left untreated may progress to irreversible lung injury and chronic lung disease [11,12]. PBB is clinically defined as the presence of an isolated chronic wet cough in the absence of pointers suggestive of alternative causes that resolves with appropriate antibiotic treatment [11,13]. CSLD is a clinical syndrome of chronic endobronchial suppuration characterised by a chronic wet cough with or without evidence of bronchiectasis on a chest high-resolution computed tomography (cHRCT) scan whereas, bronchiectasis refers to CSLD with the presence of radiological features [13]. Whether these conditions are different or reflect severity as part of a spectrum is yet to be determined.

Despite the impacts of chronic cough in children, data on the prevalence, natural history and development of chronic cough (particularly that which distinguishes between wet and dry cough) following ARI are scarce. A UK study examining the duration of acute cough in pre-school children presenting to primary care facilities found that in 10% of children cough persisted beyond 25 days [14]. A systematic review conducted by the same lead researchers reports that 10% of children were still coughing at 20–21 days [15]. Neither of these publications detailed cough type (wet or dry), the predictors of cough persistence or the outcomes (i.e. diagnosis or treatment) beyond the stated time points. A later study conducted in New Zealand however found that 74% of children <2 years of age had a history of chronic moist cough, moist cough or crackles and/or an abnormal chest x-ray 10–14 months after admission with severe lower respiratory tract infection [16]. Furthermore, there are no data detailing the types of ARIs that children present with in which a pre-existing chronic cough may be overlooked.

Inaccurate diagnosis and inappropriate investigation and management of chronic cough in children are not uncommon [17]. Misdiagnosis of asthma occurs and the diagnostic process is further complicated by the fact that the co-existence of asthma is not uncommon [11,18]. There is a clear need to improve chronic cough management in children, with guidelines now available in Australia and internationally [13,19,20]. However, these guidelines are not applicable to children presenting with ARI and associated cough to the ED setting.

EDs may be the sole primary health care provider for many families with children suffering from ARIs and associated cough and/or the alternative provider if the child does not improve after attendance at a general practitioner or other primary health care provider. A study conducted in 2012 by Liberman and colleagues [21] reported substantially low rates of primary care follow-up at both 7 and 30 days post attendance at a paediatric ED with ARI. This, in combination with the potential to progress to chronic cough, highlights the need for data that aims to inform evidence-based paediatric cough management guidelines specific to this population.

Despite the importance of cough as a cause of substantial childhood morbidity and associated economic, family and social costs, data on the aetiology and natural history of the symptom are scarce. Additionally, there are no data on the predictors of chronic cough in children following ARI. Consequently evidence-based clinical management guidelines to prevent chronic cough or to facilitate its early detection and management during and after ARI in this population are lacking. The availability of such data may assist in the development of evidence-based guidelines to improve the early detection and management of chronic cough in at-risk children during and after ARI.

### Aims and objectives

This paper aims to comprehensively describe the protocol employed to investigate the natural history, epidemiology, aetiology and outcomes of cough during and after ARI in children presenting to a tertiary paediatric ED. The data collected will potentially inform studies leading to the development of evidence-based clinical guidelines to improve the early detection and management of chronic cough in children.

The primary objective of this study is to determine the prevalence of parent reported chronic cough (≥4 weeks duration) amongst children at 28 days following presentation with ARI to a tertiary paediatric ED. Secondary objectives are:

• To determine the prevalence of parent reported chronic cough by type (wet or dry) amongst children at 28 days following presentation with ARI to a tertiary paediatric ED

• To determine the prevalence of underlying lung disease (PBB, CSLD or bronchiectasis) amongst children with ARI presenting to a tertiary paediatric ED

• To identify predictors/risk factors independently associated with chronic cough at 28 days following presentation to a tertiary paediatric ED with ARI

• To identify viral and bacterial pathogens associated with ARI at presentation to a tertiary paediatric ED

• To identify upper airway viral and bacterial pathogens associated with chronic cough at 28 days following presentation to a tertiary paediatric ED with ARI

## Methods/design

### Setting

The Royal Children’s Hospital (RCH), Brisbane, Australia, was chosen for the setting of this cohort study as it is the largest tertiary paediatric public hospital in the state. The ED services an average of 29000 children per year. Approximately 4116 (14%) presentations per year are for respiratory complaints, with 2220 (54%) of these reporting cough as a symptom (RCH, unpublished ED triage data, 2010). The ED has ready access to full diagnostic services and, amongst other services, the RCH operates a large tertiary paediatric respiratory medicine service.

### Research team

The research team responsible for inception, implementation, actioning and management of the protocol includes paediatric specialists in the fields of respiratory and ED medicine, microbiology, epidemiology, nursing and biostatistics. A team of clinical nurses from the Paediatric Emergency Research Unit (PERU) at the RCH ED are responsible for participant recruitment and baseline data collection. A study specific research assistant completes participant follow-up, data entry and attends to daily study requirements such as the booking of specialist reviews. A paediatric respiratory specialist is responsible for the review and assessment of children with chronic cough at 28 days post enrolment.

### Study design

A prospective cohort study of children aged <15 years attending the RCH ED for a respiratory illness that includes parent reported cough (wet or dry) as a symptom. Enrolled children are followed for 28 days post ED presentation to ascertain cough status following ARI. Children with persistent cough at day 28 (i.e. no more than a 3 day break in cough during the follow-up period) will undergo a respiratory assessment within 2 weeks by a paediatric respiratory physician.

The study protocol has been approved by the Queensland Children’s Health Services Human Research Ethics Committee (HREC/11/QRCH/83) and by the Medical Research Ethics Committee of the University of Queensland (2012000700). Standard operating procedures for all study operations have been developed and implemented in accordance with International Conference on Harmonisation - Good Clinical Practice [22] guidelines and the National Health and Medical Research Council’s (NHMRC) [23] ethical guidelines.

### Recruitment

Participant recruitment for the study began in December 2011 and will continue through until July 2015 to account for annual variations in ARI prevalence. Children presenting to the RCH ED with a possible respiratory illness (cough and/or fever) are identified by a PERU nurse at the time of triage (Monday-Saturday, between 0600–2100 hours) for potential participation in the study. The PERU nurse then assesses the child for eligibility through medical record review, discussion with the treating medical team and/or parent/guardian. The PERU nurse then invites eligible children and their parents to the study via the provision of child and parent specific plain language statements. Written informed consent from parents/guardians and assent from children aged ≥12 years is obtained in accordance with the Declaration of Helsinki [24] and NHMRC [23] guidelines. All children presenting to the RCH ED with a possible respiratory illness are recorded on a detailed screening log by a PERU nurse. This log contains de-identified demographic and ED triage data as well as the reason for non-participation in the study (eg; ineligible, refused, presented outside of recruitment hours).

### Inclusion criteria

Age <15 years; parent reported cough as a symptom, and; present in the ED between the hours of 6am and 9pm (or an inpatient ward within 24 hours post ED presentation).

### Exclusion criteria

Known diagnosis of chronic lung disease (excluding asthma); immunosuppressive condition; use of immunomodulating drugs (other than oral or inhaled steroids) in the 30 days prior to presentation, and; insufficient English to understand the requirements of the study.

### Study participation

**Figure 1:**
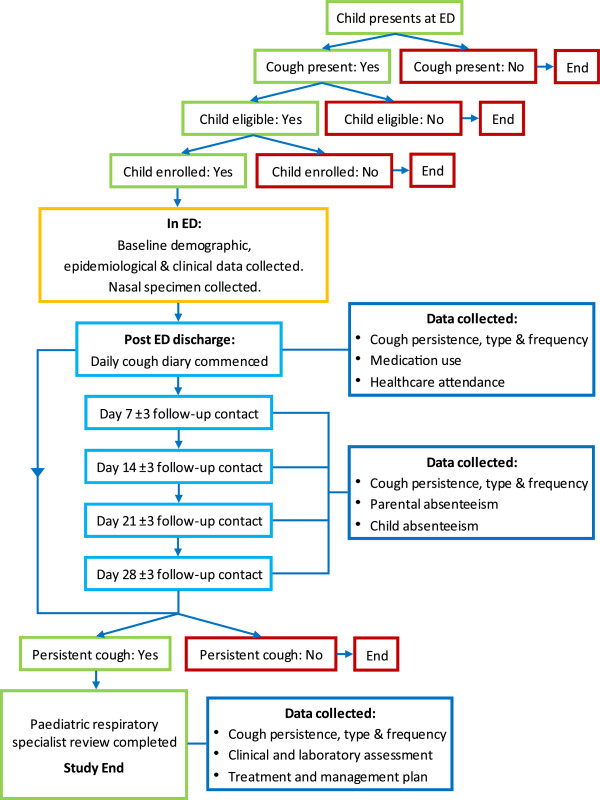
Subject participation.

Figure 1 provides an overview of subject participation. Subjects participate for 28(±3) days following initial presentation at the ED. Enrolment into the study has no bearing on the medical care provided to children in the ED which is conducted separately and in accordance with hospital policies and procedures. Parents are also encouraged to practice normal healthcare seeking behaviours throughout the duration of their participation. Children with parent reported persistent cough at day 28 (no cessation in cough for >3 consecutive days) are reviewed by a study specific paediatric respiratory physician within 2 weeks. Participation for those children ends once specialist review and any relevant investigations are complete. Children may enrol more than once provided they still satisfy inclusion and exclusion criteria.

### Procedures

Table 1 provides an overview of study procedures and data collected. At enrolment a PERU nurse completes a comprehensive questionnaire with the child and their guardian (published as an online addendum; Additional file 1). Data is obtained and recorded through parental interview, consultation with the treating medical team and through the retrieval of information from medical notes and hospital software packages. Demographic, epidemiological, and clinical data are recorded, including but not limited to; presentation symptomology, healthcare utilisation prior to ED presentation (alternative, primary and tertiary care services), current and past medical history, medication use, ED clinical management, treatment and diagnosis, clinical investigation results and socioeconomic status. Historical variables and those previously described as risk factors for paediatric ARI and lung disease will also be collected at enrolment, including; past respiratory history, familial history of atopy, asthma and lung disease, household information (e.g. number of occupants), pregnancy related factors (e.g. gestational age and birth weight), tobacco smoke exposure and being of Aboriginal or Torres Strait Islander decent. In cases where the child was admitted to a RCH ward post ED discharge, basic hospitalisation data will also be collected, including; primary and secondary diagnoses, length of stay, clinical investigation data and respiratory medicine consultations. A bilateral anterior nasal swab will be collected (or a sample of nasopharyngeal aspirate if obtained during clinical care).

**Table 1:** Outline of study procedures and data collected

	**Day 0**	**Day 7**	**Day 14**	**Day 21**	**Day 28**	**Specialist review**
Screening	**X**					
Assess eligibility criteria	**X**					
Informed consent	**X**					
Demographic data	**X**					
Epidemiological data	**X**					
Risk factor data	**X**					
Cough score, type and persistence data	**X**	**X**	**X**	**X**	**X**	**X**
Healthcare utilisation data	**X**	**X**	**X**	**X**	**X**	**X**
Medication use data	**X**	**X**	**X**	**X**	**X**	**X**
Parent and child absenteeism	**X**	**X**	**X**	**X**	**X**	**X**
Nasal swab	**X**					**X**
Clinical treatment data	**X**					**X**
Clinical investigations	**X**					**X**
Specialist review management plan						**X**
Specialist review outcomes						**X**

Parents are also asked to complete a previously validated verbal category descriptive (VCD) [25] score cough diary card for 28 days post enrolment. The recruiting PERU nurse provides the parent with both verbal and written instruction on how to complete the dairy card and a reply paid envelope to facilitate diary return. Parents also receive weekly dairy reminders via phone or email. Diary items include, cough persistence and frequency, medication use, and whether or not they sought further medical advice for the cough. If the diary card shows that the child has not stopped coughing for a period of greater than three consecutive days during the 28 day follow-up period, he/she will be referred to a study specific paediatric respiratory specialist at the RCH within 2 weeks for further investigation and management.

Follow-up contacts, via phone or email are also conducted by a study specific research assistant with parents at day 7, 14, 21 and 28(±3). Three contact attempts are made at each follow-up time point. If at any time point a contact is unsuccessful, a diary card reminder is still issued and each successive contact attempted. These contacts are used to ascertain parent reported cough persistence and type, parental absence from work due to their child’s cough, missed day-care/school due to cough and whether or not the child’s cough has ceased for a period of greater than three consecutive days during the follow-up period. In cases where a diary card is not returned, cough persistence information gathered at these contacts will identify children requiring specialist review.

At specialist review a comprehensive assessment is completed in accordance with current cough management guidelines [13,19]. Assessment items include, but are not limited to; cough persistence, frequency and type, physical examination for chest wall deformity and clubbing, chest sound auscultation, spirometry (where age appropriate) and a repeat bilateral anterior nasal swab. Other investigations such as chest imaging (x-ray or cHRCT), immunological work-up, sweat chloride testing and bronchoscopy are performed as clinically indicated. Study participation for these children ends once all relevant investigations are complete and an appropriate management or treatment plan is in place.

### Laboratory methods

Bilateral anterior nasal swabs will be stored at −80°C within 24 hours of collection and transferred to the Queensland Paediatric Infectious Diseases Laboratory, Royal Children’s Hospital, Brisbane, for viral and bacterial identification by polymerase chain reaction (PCR) testing.

Bacterial PCR testing will include *M*. *pneumonia*, *S*. *pneumoniae*, nontypeable *Haemophilus influenza* and *M*. *catarrhalis*. A 16S signature sequence will be used to detect all strains of Chlamydiales as an initial screen before positive specimens are tested for specific *C*. *trachomatis*, *C. pneumoniae* and *S*. *negevensis* sequences. PCR testing will further detect 17 viruses associated with the human respiratory tract including; adenovirus, respiratory syncytial virus, influenza virus types A & B, parainfluenza virus types 1–3, human metapneumovirus, human rhinoviruses, human coronaviruses (OC43, 229E, NL63 + HKU1), human bocavirus and human polyomaviruses KI and WU as per published methods [26-30]. Should more than one respiratory viral or bacterial pathogen simultaneously appear in respiratory specimens, quantification of individual nucleic acids may help identify the dominant agent within these classes of pathogens by comparing cycle threshold (Ct) values [31]. The dominant pathogen in each sample is identified as that with a Ct-value at least 3 cycles lower than the Ct-values for any other respiratory pathogen [31].

### Data handling and storage

Data collected over the study duration are recorded in a paper based case report form and later entered into a password protected FileMaker Pro Advanced V12 (FileMaker Inc, Santa Clara, California) database. Copies of source documents, such as investigation results, are filed in the participants individual study folder and entered into the electronic database. At participant completion a file audit is completed in which paper based and electronic records are checked for consistency, completeness and accuracy of data. Data query forms are completed for any discrepancies and returned to the appropriate study staff for correction. Following this the chief investigator reviews all participant files and approves them for signoff and storage. All study folders are stored in a locked secure cabinet. Access to identifiable participant information is only provided to immediate study staff unless otherwise required by legislative or regulatory agencies.

### Sample size

The RCH ED has an average of 343 presentations per month for respiratory complaints of which 185 (54%) have cough recorded as a symptom (RCH, unpublished ED triage data, 2010). One hundred and forty eight (80%) of these presentations occur during study recruitment hours (6am to 9pm) (RCH, unpublished ED triage data, 2010) and thus, assuming a 70% enrolment, provides a potential sample of 1243 participants per year. As the prevalence of persistent cough amongst children with ARI is unknown, an estimated prevalence of 50% was used for sample size calculation to produce the highest estimate. To address the primary objective and detect a prevalence of 50% per year (α=0.05 and 95% confidence interval) assuming a 30% loss-to-follow-up, an annual minimum of 500 children with complete data at day 28 is required. Bi-monthly reviews are completed to evaluate recruitment progression and interventions implemented where required.

### Data analysis

Descriptive analyses including demographic, clinical, laboratory and risk factor data will be tabulated and expressed as proportions and/or means of the selected characteristics with the corresponding 95% confidence intervals. Where continuous data are not normally distributed, medians with accompanying interquartile ranges will be presented. The primary analysis will be the proportion of children with persistent cough at day 28(±3) post presentation to the ED with a respiratory illness. Differences in demographic, clinical, laboratory and risk factor data between children with and without cough at day 28(±3) will be assessed using two sided t-tests for the comparison of means and chi-square tests for the comparison of proportions. Where data are not normally distributed non-parametric methods, such as a Mann–Whitney *U* test, will be used. Multivariate analyses, for example, a generalised linear model with logit link, will be performed to evaluate variables independently associated with chronic cough at day 28(±3).

Laboratory data arising from nasal swabs will be analysed for each study time point (enrolment and specialist review). Individual pathogens, their proportions and corresponding 95% confidence intervals will be tabulated and presented. These analyses will be conducted by individual pathogen and any pathogen overall and will account for variation in bacterial and/or viral load, mode of collection (nasal swab or nasopharyngeal aspiration) and the quality of specimen collection (as reported by the collecting nurse). The association between bacterial and viral co-infection and chronic cough will be explored through multiple regression models after adjusting for potential confounders.

## Discussion

This study will be the first to comprehensively describe the natural history, epidemiology, aetiology and outcomes of chronic cough, during and after ARI in children. Our prospective cohort study design in conjunction with a comprehensive clinical, epidemiological and microbiological approach will enable the collection of data that will address issues at both the time of presentation and during the recovery phase. Results will be published in national and international peer-reviewed journals and where appropriate presented at relevant conferences. We believe the dissemination of our results will contribute to future research aiming to develop and/or review new and existing evidence-based paediatric cough management guidelines.

### Rationale for study endpoints

There are limited studies that have evaluated the progression to chronic cough post ARI and none that have differentiated between wet and dry cough. Similarly there are limited data from select populations on the predictors of, and microorganisms associated with, chronic cough post ARI. The definition of chronic cough used here is a cough lasting >4 weeks. This definition is consistent with both prior research [8,14,15] and the American College of Chest Physicians guidelines for evaluating cough in children [19]. A break in cough of greater than 3 consecutive days was incorporated into our definition to ensure that children who recover but subsequently become unwell with a second cough illness are not misclassified as having chronic cough. The continued participation of children until day 28 regardless of cough persistence will elicit data on the frequency of cough reoccurrence post ARI recovery and allows a more comprehensive comparison of children who do and do not continue to cough during the follow-up period.

Furthermore, ARIs in children are commonly the result of bacterial and viral infections. However, very few children presenting to the ED (or primary care settings) with ARI undergo bacterial and viral testing. The collection of bilateral anterior nasal swabs on all study children will identify viral and bacterial pathogens associated with ARI and cough at both the time of ED presentation and specialist review. An extended panel of known respiratory pathogens (previously discussed) was chosen to ensure that pathogens excluded from routine respiratory PCR testing in the ED were included for analysis and contribute to the literature on pathogens for which the association between nasal detection and clinical illness is still being explored (e.g. polyomaviruses) [32]. Using an extended panel also enhances our ability to explore viral and bacterial interactions and their effects on cough outcomes.

### Limitations

#### Selection bias

Given funding, feasibility and logistic constraints only children present at the RCH between the hours of 6am to 9pm will be approached for participation. Children and their families attending the ED outside of these hours may differ from those attending during these hours. De-identified demographic and ED triage data will be collated and analysed to assess the extent to which children who were potentially eligible but not enrolled may bias the study findings. Furthermore, a difference may exist between families who seek care through the ED and those who attend primary care facilities.

Given that families may attend multiple healthcare facilities for management of the same illness, that many families seen in the ED are referred from other healthcare settings and that EDs are commonly used as a substitute for primary care services [33-35], the effects of restricting recruitment to a tertiary ED on the results are likely to be limited. Collection of illness severity, symptom, ED triage and prior healthcare utilisation data at enrolment will enable assessment and description of how our results may be biased. Furthermore, as recruitment is restricted to one tertiary paediatric ED in Brisbane, Queensland, Australia, the results are likely to be more representative of children living in urban communities and developed countries with temperate climates.

It is also plausible that parents of children with recurrent cough are more likely to enrol and complete the study, potentially overestimating the prevalence of chronic cough at day 28. Additionally, it is possible that children with existing undiagnosed chronic lung disease may be enrolled in the study also inflating the prevalence of chronic cough. Data pertaining to the child’s past respiratory health in conjunction with a comprehensive assessment of children with persistent cough at specialist review will allow assessment of this potential bias. It is possible these children will be amongst those referred for respiratory physician review and the identification of these children will be an important study outcome.

There is also the potential of bias derived from loss-to-follow-up, particularly as those who complete the study may differ from those who do not. Demographical, epidemiological, risk factor and clinical data collected at enrolment in combination with weekly cough persistence data will permit sensitivity analyses to assess the impact of this bias on study outcomes.

### Measurement bias

Parent proxy reporting via a diary card using a VCD score is the chosen method for obtaining cough persistence data over the follow-up period. Although it is possible to collect the same data using objective measures, for example through use of ambulatory cough meters, they are not feasible as analysis is resource heavy. Furthermore, given the intended size of our cohort it was not feasible in terms of cost or practicality. Chang et al. [25], report that VCD diary cards have the highest correlation to cough frequency when measured objectively and hence, can be used in the absence of practical objective measures to evaluate cough severity and persistence.

Parental report is also used to determine cough type over the follow-up time period. It is possible that some misclassification will occur, although this is likely to be limited. Chang et al. [36], found that parental assessment of cough type had excellent agreement with that of clinicians (K= 0.75, 95% CI: 0.58-0.93) and that clinicians' assessments were only marginally better than that of parents when compared to bronchoscopic findings. At specialist review (where required), both the parent and treating physician report on cough type allowing for the assessment of inter-rater reliability and the potential for derived bias.

Furthermore, on any given day in the ED there are numerous different clinicians with differing levels of expertise and experience. Consequently, diagnosis, clinical investigations performed and treatment initiated may vary significantly across physicians. The collection of detailed symptom, treatment and clinical data, as well as the collection of study specific anterior nasal swabs will minimise the effect of any potential bias by reducing dependence on clinician directed data. Furthermore, through employing a small team of study specific respiratory specialists from the same centre, the potential for bias derived from inconsistency of diagnosis at the time of specialist review will be minimised.

## Conclusion

Chronic cough is an under-recognised and inadequately researched cause of morbidity and decreased quality of life in children. This study will describe the natural history, epidemiology, aetiology and outcomes of chronic cough in children presenting to a tertiary paediatric ED. Our results will inform studies for evidence-based guidelines to improve the early detection, prevention and management of chronic cough in children during and after ARI.

## Abbreviations

ARI: Acute respiratory illness; cHRCT: Chest high resolution computerised tomography; Ct: Cycle threshold; CSLD: Chronic suppurative lung disease; ED: Emergency department; NHMRC: National Health and Medical Research Council; PBB: Protracted bacterial bronchitis; PCR: Polymerase chain reaction; PERU: Paediatric Emergency Research Unit; RCH: Royal Children’s Hospital, Brisbane, Australia; VCD: Verbal category descriptive.

## Competing interests

The authors declare they have no competing interests.

## Authors’ contributions

KFO and ABC conceptualised the study. All authors contributed to protocol development. ABC and JM contributed respiratory specialist advice. NP and JA contributed emergency specialist advice. TS contributed specialist microbiological advice. MD provides statistical support for the study. KFO and BJD oversee the everyday functioning of the study with support from ABC, JM, NP, JA and TS. BJD drafted the manuscript. All authors reviewed and approved the final manuscript.

## Pre-publication history

The pre-publication history for this paper can be accessed here:

http://www.biomedcentral.com/1471-2431/13/125/prepub

## Supplementary Material

Additional file 1 ED Cough Study Case Report Form. Click here for file
