# Supplementary material for: The development of chronic cough in children following presentation to a tertiary paediatric emergency department with acute respiratory illness: study protocol for a prospective cohort study
Source: BMC Pediatr. 2013 Aug 15;13:125. doi: 10.1186/1471-2431-13-125 (PMC3766129; doi:10.1186/1471-2431-13-125)
Supplement: Additional file 1 — ED Cough Study Case Report Form. [file 1471-2431-13-125-S1.pdf]

Queensland Children's Medical Research Institute

ED Cough Study Case Report Form

(Affix RCH Identification Label Here)

Study ID: **EDCS|__|__|__|__|**

Has the child been previously enrolled in this study? ☐ Yes ☐ No ☐ Unknown

CONTACT DETAILS

Who will be the primary contact parent/guardian for the purpose of this study?

☐ **Mother** ☐ **Father** ☐ **Guardian** ☐ **Other:** *Specify* ______________________________

Primary contact's full name: _____________________________________

Primary contact's contact details:

Home phone: ____

Work phone: ____

Mobile: ____

Email:____

Preferred method of contact for follow-up: ☐ Home phone
☐ Work phone
☐ Mobile
☐ Email

Preferred time of day for follow-up contact: _________am _____pm ☐ Any

Workbook completion: ☐ Emergency Department
☐ RCH Ward (CRF to be completed in full)
☐ Emergency Department and phone interview (Email Kerry-Ann O'Grady)
Reason not completed in ED: ____
Preferred contact time: ____/____/______ __ __:__ __ hrs (must be within 24 hours)

If a phone interview is required for workbook completion continue to Part A (Page 2)

PART A: ENROLMENT DETAILS

A1. DATE OF ED PRESENTATION: ___ ___ ___ ___ ___ ___ ___ ___ ___
day month year

A2. TIME OF ED PRESENTATION: __ __:__ __ **hrs (24 hour time)**

A3. INFORMED CONSENT: Has informed consent/assent been obtained and a copy given to the participant and/or parent/guardian? Yes ☐ No ☐ **Date signed:** ____/____/_____

A4. DEMOGRAPHICS

(Affix RCH Identification Label Again Here)

A5.Check Inclusion criteria again

All subjects enrolled must satisfy the following criteria at study entry:

Written informed consent/assent obtained from the subject or the parent(s) or guardian(s) of the subject.	☐ Yes ☐ No
Child aged < 15 years	☐ Yes ☐ No
Presents with respiratory illness with parent reported cough as a symptom	☐ Yes ☐ No
Present in the ED between 6am and 9pm.	☐ Yes ☐ No

A6. Exclusion criteria

If a participant meets any of the following criteria, they do not continue with the rest of the data collection

Known diagnosis of chronic lung disease (excluding asthma)<br><br>Eg. Cystic Fibrosis, Bronchiectasis, Chronic Suppurative Lung Disease, Bronchomalacia, Congenital or neonatal lung disease, Protracted Bacterial Bronchitis	☐ Yes ☐ No
Known immunosuppressive condition including HIV and cancer, leukaemia	☐ Yes ☐ No
Immunomodulating drugs in the 30 days **prior** to presentation. Inhaled steroids and oral steroids (eg prednisolone) are allowed	☐ Yes ☐ No

A7. Is the child eligible to continue participation in the study? ☐ Yes ☐ No

If a phone interview is required for workbook completion continue to Part C (Page 8)

PART B: CLINICAL HISTORY

B1. Primary reason for presentation to ED (parent report):___________________________________

B2. Triage category: ☐ 1 ☐ 2 ☐ 3 ☐ 4 ☐ 5

B3. Date first symptom of current illness started: ___ ___ (day) ___ ___ ___ (month) ___ ___ ___ ___ (year)

B4. How many days has your child had his/her current cough?

☐ < 3 ☐ 3 - <7 ☐ 7 - <14 ☐ 14 - <21 ☐ 21 - < 28 ☐ >28 ☐ Unknown

B5. Has your child had any of the following symptoms/problems in the past 7 days:

Note: present at time of presentation is to be assessed by the research nurse by both asking the parent and clinical assessment/ED notes. A "Not applicable" response is for circumstances in which the child is too young for a valid response to be possible.

	Yes	*No*	*Unk*	*N/A*	*# of days symptom was present*	*Present while in ED?*
a. Fever/temp/feel hot	☐	☐	☐	☐	|__|__| ☐ Unk	☐ Yes ☐ No ☐ N/A
b. Moist/wet/gurgly cough	☐	☐	☐	☐	|__|__| ☐ Unk	☐ Yes ☐ No ☐ N/A
c. Dry cough	☐	☐	☐	☐	|__|__| ☐ Unk	☐ Yes ☐ No ☐ N/A
d. Runny nose	☐	☐	☐	☐	|__|__| ☐ Unk	☐ Yes ☐ No ☐ N/A
e. Increased tiredness	☐	☐	☐	☐	|__|__| ☐ Unk	☐ Yes ☐ No ☐ N/A
f. Sore throat	☐	☐	☐	☐	|__|__| ☐ Unk	☐ Yes ☐ No ☐ N/A
g. Chest indrawing	☐	☐	☐	☐	|__|__| ☐ Unk	☐ Yes ☐ No ☐ N/A
h. Wheeze/whistle	☐	☐	☐	☐	|__|__| ☐ Unk	☐ Yes ☐ No ☐ N/A
i. Shortness of breath	☐	☐	☐	☐	|__|__| ☐ Unk	☐ Yes ☐ No ☐ N/A
j. Fast breathing	☐	☐	☐	☐	|__|__| ☐ Unk	☐ Yes ☐ No ☐ N/A
k. Nasal flaring	☐	☐	☐	☐	|__|__| ☐ Unk	☐ Yes ☐ No ☐ N/A
l. Earache	☐	☐	☐	☐	|__|__| ☐ Unk	☐ Yes ☐ No ☐ N/A
m. Chills	☐	☐	☐	☐	|__|__| ☐ Unk	☐ Yes ☐ No ☐ N/A
n. Muscle aches/ pains	☐	☐	☐	☐	|__|__| ☐ Unk	☐ Yes ☐ No ☐ N/A
o. Headache	☐	☐	☐	☐	|__|__| ☐ Unk	☐ Yes ☐ No ☐ N/A
p. Irritability	☐	☐	☐	☐	|__|__| ☐ Unk	☐ Yes ☐ No ☐ N/A
q. Vomiting	☐	☐	☐	☐	|__|__| ☐ Unk	☐ Yes ☐ No ☐ N/A
r. Diarrhoea*	☐	☐	☐	☐	|__|__| ☐ Unk	☐ Yes ☐ No ☐ N/A
s. Cyanosis	☐	☐	☐	☐	|__|__| ☐ Unk	☐ Yes ☐ No ☐ N/A
t. Unsettled sleep	☐	☐	☐	☐	|__|__| ☐ Unk	☐ Yes ☐ No ☐ N/A
u. Missed school/daycare	☐	☐	☐	☐	|__|__| ☐ Unk	☐ Yes ☐ No ☐ N/A
v. Unable to play/exercise	☐	☐	☐	☐	|__|__| ☐ Unk	☐ Yes ☐ No ☐ N/A
w. Feeding difficulties	☐	☐	☐	☐	|__|__| ☐ Unk	☐ Yes ☐ No ☐ N/A
x. Poor appetite	☐	☐	☐	☐	|__|__| ☐ Unk	☐ Yes ☐ No ☐ N/A
y. Stridor	☐	☐	☐	☐	|__|__| ☐ Unk	☐ Yes ☐ No ☐ N/A

*** Note: defined as ≥ 3 loose stools in a 24 hour period**

B6. I am now going to ask you to score your child's cough for both today and last night. Cough score – please let me know which one of these options best describes your child's cough.

Day-time cough score (today)	Night-time cough score (last night)
0 = No cough during the day	0 = No cough at night
1 = Cough for one-two short periods only	1a = Cough on waking only (cough did not wake child up)
2 = Cough for more than two short periods	1b = Cough on going to sleep only
3 = Frequent coughing but does NOT interfere with school or other daytime activities	2 = Awoken once or awoken early due to coughing
4 = Frequent coughing which DOES interfere with school or other daytime activities	3 = Frequent waking due to cough(s)
5 = Cannot perform most usual daytime activity due to severe coughing	4 = Frequent cough most of the night
	5 = Distressing cough

B7. Has the child been seen by other health professionals/services other than this ED presentation for his/her current illness? ☐ Yes ☐ No ☐ Unknown

B7.1 If yes, tick all that apply:

☐ GP	# times seen: |__|__|	☐ Unk	#days since last seen: |__|__|	☐ Unk
☐ Other ED/Hospital	# times seen: |__|__|	☐ Unk	#days since last seen: |__|__|	☐ Unk
☐ Community health nurse	# times seen: |__|__|	☐ Unk	#days since last seen: |__|__|	☐ Unk
☐ School nurse	# times seen: |__|__|	☐ Unk	#days since last seen: |__|__|	☐ Unk
☐ Pharmacist	# times seen: |__|__|	☐ Unk	#days since last seen: |__|__|	☐ Unk
☐ Natural therapist	# times seen: |__|__|	☐ Unk	#days since last seen: |__|__|	☐ Unk
☐ Other (Specify)________________________	# times seen: |__|__|	☐ Unk	#days since last seen: |__|__|	☐ Unk

B8. Respiratory history in past 12 months

**B8.1** In the past 12 months, has your child had a cough that has lasted more than 4 weeks?	☐ Yes ☐ No ☐ Unknown
If yes: *How many times has the child had a cough that has lasted more than 4 weeks?*	|__|__| ☐ Unknown
Did you see a doctor about this cough?	☐ Yes ☐ No ☐ Unknown
If yes, what was the diagnosis? _________________________	☐ No diagnosis ☐ Unknown
**B8.2** Has your child been diagnosed by a doctor with a respiratory illness in the past 12 months?	☐ Yes ☐ No ☐ Unknown
If yes: *Asthma/Reactive airways disease*	☐ Yes ☐ No # times |__|__|
Pneumonia (confirmed by chest xray)	☐ Yes ☐ No # times |__|__|
Influenza (confirmed by laboratory test, eg NPA or blood test)	☐ Yes ☐ No # times |__|__|
Bronchiolitis	☐ Yes ☐ No # times |__|__|
Bronchitis	☐ Yes ☐ No # times |__|__|
Croup	☐ Yes ☐ No # times |__|__|
Whooping cough (laboratory confirmed)	☐ Yes ☐ No # times |__|__|
Otitis media	☐ Yes ☐ No # times |__|__|
Tonsillitis	☐ Yes ☐ No # times |__|__|
Other	☐ Yes ☐ No # times |__|__|
*Specify*____	
**B8.3** Has your child been hospitalised for an acute respiratory illness in the past 12 months?	☐ Yes ☐ No ☐ Unknown
If yes: Asthma /Reactive airways disease	☐ Yes ☐ No # times|__|__|
Pneumonia	☐ Yes ☐ No # times|__|__|
Influenza	☐ Yes ☐ No # times|__|__|
Bronchiolitis	☐ Yes ☐ No # times|__|__|
Bronchitis	☐ Yes ☐ No # times|__|__|
Whooping Cough (laboratory confirmed)	☐ Yes ☐ No # times|__|__|
Croup	☐ Yes ☐ No # times|__|__|
*Other (specify)*______________________________________	☐ Yes ☐ No # times|__|__|
**B8.4** Does your child have any allergies? *If yes:*	☐ Yes ☐ No ☐ Unknown
Dust ☐ Yes ☐ No	
Pollen ☐ Yes ☐ No	
Insects ☐ Yes ☐ No	
Medications ☐ Yes ☐ No *Specify*___________________________	
Food ☐ Yes ☐ No *Specify*___________________________	
Other: ☐ Yes ☐ No *Specify*___________________________	

**B8.5**. Has your child ever had an itchy rash that was coming and going for at least 6 months?	☐ Yes ☐ No ☐ Unknown
If yes, a) has your child had this rash in the past 12 months?	☐ Yes ☐ No ☐ Unknown
b)Has this itchy rash at any time affected any of the following places: the folds of the elbows, behind the knees, in front of the ankles, under the buttocks, or around the neck, ears or eyes?	☐ Yes ☐ No ☐ Unknown
Has your child ever had eczema?	☐ Yes ☐ No ☐ Unknown
**B8.6.** Has your child had wheezing or whistling in the chest in the last 12 months?	☐ Yes ☐ No ☐ Unknown
If no, has your child ever had wheezing or whistling in the chest?	☐ Yes ☐ No ☐ Unknown

B9. Risk factors

I am now going to ask you some questions about things that are thought to play an important role in children getting chest infections and chronic cough. We need to find out these things so we can work out what things we need to address to help prevent children from developing chronic cough

**Risk factor data**	
**9.1 Household information**	
9.1a Number of children usually living in house with your child:	|__|__| ☐ Unknown
If other children record ages of other children living in house: Child # 1____ # 2_____ #3_____ #4_______ #5________	
9.1b Number of bedrooms in the house your child usually lives in:	|__|__| ☐ Unknown
9.1c Total number people usually living in house with child:	|__|__| ☐ Unknown
**9.2 Pregnancy related factors**	
9.2a Birth weight (gms):	|__|__|__|__| ☐ Unknown
9.2b Gestational age:	_________ weeks ☐ Unknown
9.2c Maternal smoking in pregnancy:	☐ Yes ☐ No ☐ Unk
**9.3 Breastfeeding**	
9.3a Currently breastfed?	☐ Yes ☐ No ☐ Unknown
If yes, ☐ Exclusively ☐ Partially	
9.3b If no, ever breastfed?	☐ Yes ☐ No ☐ Unknown
If yes, age feeding ceased:_______________	
**9.4 Exposure to tobacco smoke**	
9.4a Current smoker?	☐ Yes ☐ No ☐ Unknown ☐ N/A
If yes, age started: ________ #/day: |__|__|	
9.4b Ever smoked?	☐ Yes ☐ No ☐ Unknown ☐ N/A
If yes, age started: ______ age stopped: ______ #day|__|__|	
9.4c Household exposure?	☐ Yes ☐ No ☐ Unknown
If yes: ☐ Mother ☐ Father ☐ Others	
**9.5 School/Childcare attendance**	
9.5a Attends school/preschool?	☐ Yes ☐ No ☐ Unknown
If yes, current class year: |__|__|	
9.5b Attends childcare:	☐ Yes ☐ No ☐ Unknown
If yes, # days per week: |__|__|	

**9.6 Family history**	
9.6a Family history of asthma/reactive airways disease?	☐ Yes ☐ No ☐ Unknown
9.6b Family history of other lung disease?<br>*If yes, specify:* ________________________________	☐ Yes ☐ No ☐ Unknown
9.6c Family history of allergies?	☐ Yes ☐ No ☐ Unknown
**9.7 Pets**<br>Does the child/child's household have any pets?<br>*If yes:*	☐ Yes ☐ No ☐ Unknown

Dog	☐ Yes ☐ No ☐ Unknown
Cat	☐ Yes ☐ No ☐ Unknown
Bird	☐ Yes ☐ No ☐ Unknown
Rabbit	☐ Yes ☐ No ☐ Unknown
Guinea Pig	☐ Yes ☐ No ☐ Unknown
Reptiles	☐ Yes ☐ No ☐ Unknown
Rabbit	☐ Yes ☐ No ☐ Unknown
Chickens/ducks	☐ Yes ☐ No ☐ Unknown
Other (specify)	☐ Yes ☐ No ☐ Unknown

**9.8 Travel**<br>Has your child travelled in the past 14 days?<br>If yes: ☐ within QLD<br>☐ Interstate (specify) __________________<br>☐ Overseas (specify) ___________________<br>Length of time away (days) ____________________<br>Date of return to home: ___/___/___	☐ Yes ☐ No ☐ Unknown

B10. Medications: Has the child been given any medications in the past 7 days, including today, and before he/she was brought to the ED. *Note: record all current antibiotics, antipyretics, anti-inflammatory, inhalants, cough and cold medications* ☐ Yes ☐ No ☐ Unknown

Generic name	Total daily dose & route	Start date	Stop date or write 'ongoing'	Indication

B10.1 Has the child been given any oral steroids, including today, and before he/she was brought to the ED in the past 30 days? ☐ Yes ☐ No ☐ Unknown

Generic name	Number of days given	Start date	Stop date or write 'ongoing'	Indication

B11. Influenza vaccination
Has the child received an influenza vaccine in the preceding 12 months?
☐ Yes, ☐ No ☐ Unknown ☐ Not applicable as aged < 6 months

Researcher Initials: ______________________ **Date: ____/____/____**

B12. Childhood immunisation schedule
Is the child age appropriately immunised?
☐ Yes, ☐ parent report ☐ confirmed by medical record ☐ confirmed by immunisation record
☐ No ☐ Unknown

B13. Socio-economic variables
I am now going to ask you some questions about your educational status, employment status, and household income. These factors are known to be important contributors to the health of children and the risk of getting chronic lung problems. These questions are also important as they help us determine how well the children that are enrolled in our study represent the broader community of Australian children and hence decide whether the results of our study can be applied to the majority of Australian children who have cough. The questions are the same questions that are asked in the Australian Census. The information will not be seen by anyone other than the researchers and will be stored securely. You can choose not to answer the questions if that is what you prefer. You can also answer the questions on this page by ticking the boxes yourself if that is more comfortable for you.

13.1a Employment (labour force) status of parent/guardian:

	Mother	Father	Guardian
Employed full time	☐	☐	☐
Employed part time	☐	☐	☐
Employed casual	☐	☐	☐
Not in paid employment	☐	☐	☐
Not applicable	☐	☐	☐
Unknown	☐	☐	☐
Declined to answer	☐	☐	☐

13.2b Total annual **household** income (gross/before tax):

<$26,000	☐
$26,000 - < $52,000	☐
$52,000 - < $78,000	☐
$78,000 - < $104,000	☐
$104,000 - <$156,000	☐
$156,000 - <$200,000	☐
≥ $200,000	☐
Unknown	☐
Declined to answer	☐

13.3c Highest education level of parent(s)/guardian

	Mother	Father	Guardian
Did not finish high school	☐	☐	☐
High school	☐	☐	☐
Certificate	☐	☐	☐
Diploma	☐	☐	☐
Bachelor degree	☐	☐	☐
Post graduate degree	☐	☐	☐
Not applicable	☐	☐	☐
Unknown	☐	☐	☐
Declined to answer	☐	☐	☐

I am now going to ask you about your child's Indigenous status. It is now a national standard for this information to be collected in all health and health research data collections. This is so we can get much better information about the health of Indigenous Australians. Indigenous Australians also have much higher rates of respiratory infections and chronic lung disease than non-Indigenous Australians. You can choose not to answer the question if you don't want to.

13.1d Is your child of Aboriginal and/or Torres Strait Islander origin?
☐ Yes, Aboriginal ☐ Yes, Torres Strait Islander ☐ Yes, Aboriginal and Torres Strait Islander
☐ No, not Aboriginal no Torres Strait Islander ☐ Not known ☐ Declined to answer

That is the end of the questions we need to ask you about you and your child. We will now take the nose swab and the rest of the information we need shall be collected from your medical record. I will also show you how to complete the diary card. NOTE: If the child has had a nasopharyngeal aspirate performed, an additional nose swab is not required so long as a sample of the aspirate is obtained (ie swab dipped into aspirated fluid).

PART C: Specimen collection

C1. Has an anterior nasal swab been collected as per protocol?

Yes, both nares ☐

No, one nare only ☐

No, NPA sample collected instead ☐

No specimens collected ☐

*C1a Reason only one nare or none collected:*____

C2. Time specimen collected: ____:_____hrs

Was the specimen collected on the ward? ☐Yes ☐No

If yes, is the child currently on antibiotic/antiviral therapy? ☐Yes ☐No

If yes please provide the generic name, dose, route and number of doses given since ED presentation:

Generic Name	Dose	Route	Number of doses given
______________________	______________________	______________________	______________________
______________________	______________________	______________________	______________________

Specimen ID number: EDCS|__|__|__|__|Day 0

C3 Record quality of specimen collection technique

	Left nare	Right nare
Good (ie tip inserted 1cm , turned 4 times against internal nare)	☐	☐
Fair (tip partially inserted or complete swabbing (4 turns) not achieved	☐	☐
Poor (difficulty in collection and unlikely to have obtained good/fair sample)	☐	☐
Not done	☐	☐
Not applicable (NPA sample collected instead)	☐	☐

C3a Comments on specimen collection: ____

____

SPECIMEN CHECK LIST

☐ Specimen labelled correctly and specimen tracking log completed

☐ Specimen placed in study box in ED

DIARY CARD CHECK LIST

☐ Instructions on completing card given to parent

☐ Child's study ID recorded on diary card

If a phone interview is required for workbook completion continue to Part D (Page 9)

PART D: Clinical information and management

D1. OBSERVATIONS/VITAL SIGNS AT TIME OF ED PRESENTATION...If any were not performed then measurements are to be performed by research staff.

a. Temperature: _______ . ____ °C Temperature method: Oral ☐ Per Axilla ☐ Aural ☐

b. Heart rate: ______________beats per minute **c.** Respiratory rate: _____________ per minute

d. O_2 Saturation (pulse oximetry – room air): ___________% **e.** On oxygen therapy? ☐ **Yes** ☐ **No**

f. Weight: ________.____kg **g.** Height: ________cms

h. Abnormal chest sounds on auscultation **as recorded by ED doctor** examining child:

☐ Yes ☐ No ☐ Unknown ☐ Not done ☐ Not reliable given child distress

If yes,

Crackles ☐ Yes ☐ No ☐ Unknown

Wheeze ☐ Yes ☐ No ☐ Unknown

Other ☐ Yes ☐ No ☐ Unknown *Specify:* ______________________________

i. Was an ENT exam **performed by the ED doctor**? ☐ Yes ☐ No ☐ Unk

If yes, ☐ Normal ☐ Abnormal ☐ Unknown ☐ Not specified

If abnormal, specify abnormality: ____

D2: Were any investigations performed whilst in the ED? ☐ Yes ☐ No ☐ Unknown
If yes, complete table

FBC	☐ Yes ☐ No ☐ Unknown	Copy of lab result attached: ☐ Yes ☐ No → ☐ Follow-up
UEC	☐ Yes ☐ No ☐ Unknown	Copy of lab result attached: ☐ Yes ☐ No → ☐ Follow-up
CRP	☐ Yes ☐ No ☐ Unknown	Copy of lab result attached: ☐ Yes ☐ No → ☐ Follow-up
NPA	☐ Yes ☐ No ☐ Unknown	Results to be followed up: ☐ Yes
Blood Culture	☐ Yes ☐ No ☐ Unknown	Results to be followed up: ☐ Yes
Blood Gases	☐ Yes ☐ No ☐ Unknown	Copy of lab result attached: ☐ Yes ☐ No → ☐ Follow-up
Chest XRay	☐ Yes ☐ No ☐ Unknown	Copy of radiology report attached: ☐ Yes ☐ No → ☐ Follow-up
Lumbar Puncture	☐ Yes ☐ No ☐ Unknown	Results to be followed up: ☐ Yes
Other (Specify)	☐ Yes ☐ No ☐ Unknown	Copy of results attached: ☐ Yes ☐ No → ☐ Follow-up

Comments:____

____

____

D3. Were any medications given in the ED? ☐ Yes ☐ No ☐ Unknown *If yes, complete information below*

Generic name	Dose	Route	Time first dose given in ED	Total doses given in ED

If a phone interview is required for workbook completion continue to Part E (below)

PART E: Emergency Department diagnosis and discharge

E1: Discharge date: ____/____/____ Discharge time: ___:___hrs

E2: ED Primary medical diagnosis at time of discharge/transfer from department (EDIS):

____

E2.1: Specify whether the primary diagnosis was provisional or confirmed
☐ Provisional ☐ Confirmed ☐ Not stated

E3: ED Secondary Medical Diagnoses at time of discharge/transfer from department:

E3a:____ ☐ Provisional ☐ Confirmed ☐ Not stated

E3b: ____ ☐ Provisional ☐ Confirmed ☐ Not stated

E3c: ____ ☐ Provisional ☐ Confirmed ☐ Not stated

E4: Was the child reviewed by a Respiratory Physician/Fellow/Registrar in the ED? ☐ Yes ☐ No ☐ Unk

If yes, specify doctor: ____

E5. Discharge destination

☐ Home, no further review
☐ Home, review in ED: *Planned review date:* ____/____/____
☐ Home, referral to/review by GP
☐ Home, referral to/review by RCH Specialist Outpatients: *Review date:* ____/____/____
☐ Admitted to RCH: *Admission date:*____/___/____ *Admission time*:___:___hrs *Ward*: ___________
☐ Transfer to other hospital: *Specify*__________________________________
☐ Did not wait for ED treatment/management

Subject Number: **EDCS** |__|__|__|__|

E6. Was the child discharged on any medications? ☐ Yes ☐ No ☐ Unknown
If yes, complete table below

Generic name	Dose	Route	Daily Frequency	Number of days

STUDY CHECKLIST

☐ Ensure the child and/or parent has a copy of the signed consent form/ information sheet & diary card.

☐ Follow-up phone calls discussed with parent/guardian & diary card explained

☐ Screening log completed

☐ Specimen log completed

☐ Post discharge CRF completion required?
If yes, CRF completion request sent: ☐ Yes
CRF completed: ☐ Yes

☐ Follow-up for outstanding results of investigations required?
If yes, log completed: ☐ Yes

☐ Follow-up for outcomes of hospital admission required?
If yes, log completed: ☐ Yes

Notes/Comments:

____

____

____

____

____

____

THIS PAGE IS INTENTIONALLY BLANK

Subject Number: **EDCS |__|__|__|__|**

Queensland Children's Medical Research Institute

ED Cough Study Case Report Form: Day 7 Follow-up

Contact attempt 1
Date: ___/___/___ Time: ___:___hrs Contact method: ☐ Phone ☐ SMS ☐ Email

Contact successful (ie parent responds to message/call): ☐ Yes ☐ No

Contact attempt 2
Date: ___/___/___ Time: ___:___hrs Contact method: ☐ Phone ☐ SMS ☐ Email

Contact successful (ie parent responds to message/call): ☐ Yes ☐ No

Contact attempt 3
Date: ___/___/___ Time: ___:___hrs Contact method: ☐ Phone ☐ SMS ☐ Email

Contact successful (ie parent responds to message/call): ☐ Yes ☐ No

1. Follow-up information

1. Does the child have a cough today? ☐ Yes ☐ No ☐ Unknown

2. If yes, what type of cough is it? ☐ Wet ☐ Dry ☐ Both ☐ Unknown

2a) Have you missed work because of this cough this week? ☐ Yes ☐ No ☐ Unknown

If yes, number of days missed work: |__|__| days

2b) Has your child missed school/childcare this week because of this cough? ☐ Yes ☐ No ☐ Unknown

If yes, number of days missed school: |__|__| days

2. What is your child's cough score for today and for last night (circle appropriate score?

Day-time cough score (today)	Night-time cough score (last night)
0 = No cough during the day	0 = No cough at night
1 = Cough for one-two short periods only	1a = Cough on waking only (cough did not wake child up)
2 = Cough for more than two short periods	1b = Cough on going to sleep only
3 = Frequent coughing but does NOT interfere with school or other daytime activities	2 = Awoken once or awoken early due to coughing
4 = Frequent coughing which DOES interfere with school or other daytime activities	3 = Frequent waking due to cough(s)
5 = Cannot perform most usual daytime activity due to severe coughing	4 = Frequent cough most of the night
	5 = Distressing cough

Has your child stopped coughing for 3 or more days in the past week? ☐ Yes ☐ No ☐ Unknown

Parent reminded to continue daily diary card and to record any medications given: ☐ Yes ☐ No

Parent reminded of next follow-up contact at day 14 (± 3 days) ☐ Yes ☐ No

Comments

____

____

____

Researcher Initials: ______________________ **Date: ____/____/____**

Subject Number: **EDCS |__|__|__|__|**

ED Cough Study Case Report Form: Day 14 Follow-up

Contact attempt 1
Date: ___/___/___ Time: ___:___hrs Contact method: ☐ Phone ☐ SMS ☐ Email

Contact successful (ie parent responds to message/call): ☐ Yes ☐ No

Contact attempt 2
Date: ___/___/___ Time: ___:___hrs Contact method: ☐ Phone ☐ SMS ☐ Email

Contact successful (ie parent responds to message/call): ☐ Yes ☐ No

Contact attempt 3
Date: ___/___/___ Time: ___:___hrs Contact method: ☐ Phone ☐ SMS ☐ Email

Contact successful (ie parent responds to message/call): ☐ Yes ☐ No

Follow-up information
1. Does the child have a cough today? ☐ Yes ☐ No ☐ Unknown

2. If yes, what type of cough is it? ☐ Wet ☐ Dry ☐ Both ☐ Unknown
   2a) Have you missed work because of this cough this week? ☐ Yes ☐ No ☐ Unknown
   If yes, number of days missed work: |__|__| days

   2b) Has your child missed school/childcare this week because of this cough? ☐ Yes ☐ No ☐ Unknown
   If yes, number of days missed school: |__|__| days

B6. What is your child's cough score for today and for last night (circle appropriate score?

Day-time cough score (today)	Night-time cough score (last night)
0 = No cough during the day	0 = No cough at night
1 = Cough for one-two short periods only	1a = Cough on waking only (cough did not wake child up)
2 = Cough for more than two short periods	1b = Cough on going to sleep only
3 = Frequent coughing but does NOT interfere with school or other daytime activities	2 = Awoken once or awoken early due to coughing
4 = Frequent coughing which DOES interfere with school or other daytime activities	3 = Frequent waking due to cough(s)
5 = Cannot perform most usual daytime activity due to severe coughing	4 = Frequent cough most of the night
	5 = Distressing cough

Has your child stopped coughing for 3 or more days in the past week? ☐ Yes ☐ No ☐ Unknown

Parent reminded to continue daily diary card and to record any medications given: ☐ Yes ☐ No

Parent reminded of next follow-up contact at day 21 (± 3 days) ☐ Yes ☐ No

Comments
____

____

Queensland Children's Medical Research Institute

ED Cough Study Case Report Form: Day 21 Follow-up

Contact attempt 1
Date: ___/___/___ Time: ___:___hrs Contact method: ☐ Phone ☐ SMS ☐ Email

Contact successful (ie parent responds to message/call): ☐ Yes ☐ No

Contact attempt 2
Date: ___/___/___ Time: ___:___hrs Contact method: ☐ Phone ☐ SMS ☐ Email

Contact successful (ie parent responds to message/call): ☐ Yes ☐ No

Contact attempt 3
Date: ___/___/___ Time: ___:___hrs Contact method: ☐ Phone ☐ SMS ☐ Email

Contact successful (ie parent responds to message/call): ☐ Yes ☐ No

Follow-up information
1. Does the child have a cough today? ☐ Yes ☐ No ☐ Unknown

2. If yes, what type of cough is it? ☐ Wet ☐ Dry ☐ Both ☐ Unknown
   2a) Have you missed work because of this cough this week? ☐ Yes ☐ No ☐ Unknown
   If yes, number of days missed work: |__|__| days

   2b) Has your child missed school/childcare this week because of this cough? ☐ Yes ☐ No ☐ Unknown
   If yes, number of days missed school: |__|__| days

B6. What is your child's cough score for today and for last night (circle appropriate score?

Day-time cough score (today)	Night-time cough score (last night)
0 = No cough during the day	0 = No cough at night
1 = Cough for one-two short periods only	1a = Cough on waking only (cough did not wake child up)
2 = Cough for more than two short periods	1b = Cough on going to sleep only
3 = Frequent coughing but does NOT interfere with school or other daytime activities	2 = Awoken once or awoken early due to coughing
4 = Frequent coughing which DOES interfere with school or other daytime activities	3 = Frequent waking due to cough(s)
5 = Cannot perform most usual daytime activity due to severe coughing	4 = Frequent cough most of the night
	5 = Distressing cough

Has your child stopped coughing for 3 or more days in the past week? ☐ Yes ☐ No ☐ Unknown

Parent reminded to continue daily diary card and to record any medications given: ☐ Yes ☐ No

Parent reminded of next follow-up contact at day 28 (± 3 days) ☐ Yes ☐ No

Comments
____

____

Subject Number: **EDCS |__|__|__|__|__|**

ED Cough Study Case Report Form: Day 28 Follow-up

Contact attempt 1
Date: ___/___/___ Time: ___:___hrs Contact method: ☐ Phone ☐ SMS ☐ Email

Contact successful (ie parent responds to message/call): ☐ Yes ☐ No

Contact attempt 2
Date: ___/___/___ Time: ___:___hrs Contact method: ☐ Phone ☐ SMS ☐ Email

Contact successful (ie parent responds to message/call): ☐ Yes ☐ No

Contact attempt 3
Date: ___/___/___ Time: ___:___hrs Contact method: ☐ Phone ☐ SMS ☐ Email

Contact successful (ie parent responds to message/call): ☐ Yes ☐ No

Follow-up information
Does the child have a cough today? ☐ Yes ☐ No ☐ Unknown

If yes, what type of cough is it? ☐ Wet ☐ Dry ☐ Both ☐ Unknown
2a) Have you missed work because of this cough this week? ☐ Yes ☐ No ☐ Unknown
If yes, number of days missed work: |__|__| days

2b) Has your child missed school/childcare this week because of this cough? ☐ Yes ☐ No ☐ Unknown
If yes, number of days missed school: |__|__| days

B6. What is your child's cough score for today and for last night (circle appropriate score?

Day-time cough score (today)	Night-time cough score (last night)
0 = No cough during the day	0 = No cough at night
1 = Cough for one-two short periods only	1a = Cough on waking only (cough did not wake child up)
2 = Cough for more than two short periods	1b = Cough on going to sleep only
3 = Frequent coughing but does NOT interfere with school or other daytime activities	2 = Awoken once or awoken early due to coughing
4 = Frequent coughing which DOES interfere with school or other daytime activities	3 = Frequent waking due to cough(s)
5 = Cannot perform most usual daytime activity due to severe coughing	4 = Frequent cough most of the night
	5 = Distressing cough

Has your child stopped coughing for 3 or more days in the past week? ☐ Yes ☐ No ☐ Unknown

Child requires review by paediatric respiratory physician in next 2 weeks? ☐ Yes ☐ No

If yes: Appointment booked: ☐ Yes
Date of appointment: ___/___/___
Time of appointment: ___:___hrs
Physician: ☐ Dr Marchant ☐ Dr Butain ☐ Prof Chang ☐ Other (specify)________________________

Parents notified of appointment ☐ Yes → Date:___/___/___ (☐ Phone ☐ Email ☐ Letter)

Referral letter completed and signed ☐ Yes → Date:___/___/___

Comments

____

____

____

____

____

____

____

____

____

____

____

____

____

____

____

Researcher Initials: ________________________ **Date: ____/____/____**

Subject Number: **EDCS** |__|__|__|__|__|

Queensland Children's Medical Research Institute

ED Cough Study Case Report Form: Hospitalisation Record

Complete for children admitted to RCH from the ED department

(Affix RCH Identification Label Here)

H1. Admission date:____/___/____ H2. Admission time:___:___hrs H3. Admission ward: __________

H4. Discharge date:____/___/____ H5. Discharge time:___:___hrs

H6. Was the child admitted to the Intensive Care Unit during hospital admission? ☐ Yes ☐ No ☐ Unknown

H7. Was the child reviewed by the Paediatric Respiratory Team during admission? ☐ Yes ☐ No ☐ Unknown

H8. Discharge destination:
☐ Home
☐ Died
☐ Transfer to other hospital/health care facility
☐ Left without medical discharge
☐ Not stated

H9. Primary discharge diagnosis: ___________________________________ ICD-10-AM Code:________________

H10. Secondary discharge diagnoses
a) ___________________________________ ICD-10-AM Code:________________
b) ___________________________________ ICD-10-AM Code:________________
c) ___________________________________ ICD-10-AM Code:________________
d) ___________________________________ ICD-10-AM Code:________________

H11. Was a CXR performed during hospitalisation? ☐ Yes ☐ No ☐ Unknown
If yes, diagnosis: ____

H12. Was a Chest CT scan performed during hospitalisation? ☐ Yes ☐ No ☐ Unknown
If yes, diagnosis: ____

Comments:
____
____
____

Researcher Initials: ______________________ **Date:** ____/____/____

THIS PAGE IS INTENTIONALLY BLANK

Subject Number: **EDCS** |__|__|__|__|__|

ED Cough Study Case Report Form: Specialist review

(Affix RCH Identification Label Here)

Date of review: ___/___/___ Specialist performing review: ______________________

SR 1. Cough History

**SR1.1 Does the child have a cough today?**	☐ Yes ☐ No ☐ Unknown
If yes: a) how long has the child had the cough?	|__|__| weeks
b) what type of cough does the child have today?	☐ Wet ☐ Dry ☐ Variable
c) has there been ≥ 3 days without a cough since day 28?	☐ Yes ☐ No ☐ Unknown
If no, a) when was the last day of cough after day 28	____/_____/_____
b) how many days has the child NOT had a cough since day 28 of the study (ie. last day of the diary card)	|__|__| days
**SR1.2** Has the child ever had episodes of cough that have lasted more than 4 weeks?	☐ Yes ☐ No ☐ Unknown
If yes, at what age did the child first have an extended episode of cough	|__|__| ☐ Unknown

SR1.3 Cough Score for today

Day-time cough score (today)	Night-time cough score (last night)
0 = No cough during the day	0 = No cough at night
1 = Cough for one-two short periods only	1a = Cough on waking only (cough did not wake child up)
2 = Cough for more than two short periods	1b = Cough on going to sleep only
3 = Frequent coughing but does NOT interfere with school or other daytime activities	2 = Awoken once or awoken early due to coughing
4 = Frequent coughing which DOES interfere with school or other daytime activities	3 = Frequent waking due to cough(s)
5 = Cannot perform most usual daytime activity due to severe coughing	4 = Frequent cough most of the night
	5 = Distressing cough

**SR 1.4 Chest sounds/breathing difficulty**	
a) Wheeze	☐ Yes ☐ No ☐ Unknown
b) Rattle	☐ Yes ☐ No ☐ Unknown
c) Shortness of breath	☐ Yes ☐ No ☐ Unknown
d) Stridor	☐ Yes ☐ No ☐ Unknown
e) Other (Specify)______________________________	☐ Yes ☐ No ☐ Unknown

SR 2. Physical Examination

Height: |__|__|__|cm	Weight: |__|__|__|.|__|kg
Respiratory rate: |__|__|__| rpm	Increased work of breathing: ☐ Yes ☐ No ☐ Unknown<br>If yes, describe:
Cough heard: ☐ Yes ☐ No ☐ Unknown<br><br>If yes, cough type: ☐ Wet ☐ Dry	
Clubbing	☐ Yes ☐ No ☐ Unknown
Harrison sulcus	☐ Yes ☐ No ☐ Unknown
CWD	☐ Yes ☐ No ☐ Unknown
Crepitations	☐ Yes ☐ No ☐ Unknown
Wheeze	☐ Yes ☐ No ☐ Unknown
Other chest sounds<br>**If yes, specify:**	☐ Yes ☐ No ☐ Unknown
ENT Examination<br>**If abnormal, specify:**	☐ Normal ☐ Abnormal ☐ Not done
Spirometry<br>Pre B2 Loop Shape<br>Post B2 Loop Shape	 ☐ Normal ☐ Obstructive ☐ Restrictive ☐ Not done<br>☐ Normal ☐ Obstructive ☐ Restrictive ☐ Not done
Chest xray<br>**If abnormal, specify:**	☐ Normal ☐ Abnormal ☐ Not done

SR3: Specimen collection

SR3.1 Has an anterior nasal swab been collected as per protocol?

Yes, both nares ☐ Time specimen collected: ____:_____hrs

No, one nare only ☐

No specimens collected ☐

*Reason only one nare or none collected:*____

Specimen ID number: EDCS|__|__|__|__|Day ______

SR3.3 Record quality of specimen collection technique

	Left nare	Right nare
Good (ie tip inserted 1cm, turned 4 times against internal nare)	☐	☐
Fair (tip partially inserted or complete swabbing (4 turns) not achieved	☐	☐
Poor (difficulty in collection and unlikely to have obtained good/fair sample)	☐	☐
Not done	☐	☐

SR3.4 Comments on specimen collection: ____

Researcher Initials: ________________________ Date: ____/____/____

SPECIMEN CHECK LIST

☐ Specimen labelled correctly and specimen tracking log completed
☐ Specimen placed in study box in ED fridge?

SR 4. Diagnoses

SR4.1 Provisional primary diagnosis: ____

SR4.2 Comorbidities: a) ______________________ b) __________________ c) ________________________

SR4.3 Other investigations planned: ☐ HRCT ☐ Bronchoscopy ☐ Other (specify) ___________________________

SR4.4 Final primary diagnosis: ________________________________ Date confirmed: ____/_____/_____

SR4.5 Final additional diagnoses: ______________________________Date confirmed: ____/_____/_____

SR 5. Comments (including relevant management)

____

____

____

____

____

____

____

____

____

____

____

____

____

____

____

Study completion

SC1. Date of last study contact with child: ___/____/_____

SC2. Was the child withdrawn from the study? ☐ Yes ☐ No

SC2.1 If yes, specify reason for withdrawal:

☐ Protocol violation

☐ Loss to follow-up

☐ Parent withdrew consent

☐ Child withdrew consent

SC3. Has the child's diary card been returned? ☐ Yes ☐ No

SC3.1 If no, specify reason

☐ Loss to follow-up

☐ Diary card lost in mail

☐ Other (specify) ____

SC4. Have nasal specimens for this child been received at Qpid? ☐ Yes ☐ No

SC4.1 If yes, specify the specimens received: ☐ Day 0 ☐ Specialist review

SC4.2 If no, specify reason

☐ Loss to follow-up

☐ Specimen lost in transit

☐ Specimen not collected (tick specimen not collected): ☐ Day 0 ☐ Specialist review

☐ Other (specify) ____

SC5. If applicable, did the child present for specialist review? ☐ Yes ☐ No ☐ Not applicable

SC5.1 If no, specify reason
☐ Loss to follow-up

☐ Moved away from area and unable to attend

☐ Parent decision not to attend

☐ Other (specify) ____
